# Elevated Middle Cerebral Artery Peak Systolic Velocity in a Nonanemic Fetus with Alpha-Thalassemia Trait

**DOI:** 10.1155/2009/819380

**Published:** 2009-11-15

**Authors:** Kent Heyborne

**Affiliations:** Swedish Medical Center, Denver, CO 80113, USA

## Abstract

*Background*. Elevated middle cerebral artery peak systolic velocity (MCA-PSV) has been reported in nonanemic fetuses following fetal transfusion, and has been attributed to a major population of red blood cells (RBCs) with an adult mean corpuscular volume (MCV) in the fetal circulation. Reported here is an analogous case of elevated MCA-PSV with a normal fetal hematocrit and relative fetal microcytosis due to fetal *α*-thalassemia trait. *Case*. Ultrasound findings concerning for early hydrops prompted measurement of MCA-PSV, which was elevated. Cordocentesis revealed fetal microcytosis with a normal hematocrit which proved to be due to fetal *α*-thalassemia trait inherited from the mother. *Conclusion*. This case provides another example of elevated MCA-PSV with normal hematocrit and microcytosis, here due to fetal *α*-thalassemia trait. This finding provides support for the observation that MCA-PSV may be influenced by hematological indices other than the fetal hematocrit.

## 1. Introduction

Measurement of the middle cerebral artery peak systolic velocity (MCA-PSV) has become the mainstay for the detection of fetal anemia. It has been shown that MCA-PSV measurements may be increased in nonanemic fetuses with a significant population of adult RBC following multiple fetal transfusions [1–3]. While the sensitivity of the PSV measurement remains high, the false-positive rate may increase with an increasing number of transfusions. We present here an analogous circumstance of elevated MCA-PSV with a normal hematocrit due to relative fetal microcytosis secondary to *α*-thalassemia trait.

## 2. Case

The patient was a 36-year-old gravida 2 para 1 woman of Filipino descent with dichorionic twins following in vitro fertilization and embryo transfer. She was noted to have a microcytic anemia at her first prenatal visit (hematocrit 27.3%, MCV 72 fL). Hemoglobin electrophoresis was consistent with *α*-thalassemia trait. Her husband did not have a hemoglobinopathy. An ultrasound at 13 weeks 4 days gestation revealed modestly elevated nuchal thickness (NT) measurements of 3.5 and 3.3 mm for twins A and B, respectively, prompting amniocentesis at 16 weeks gestation. Normal 46, XX karyotypes were found for both twins. Due to the twin gestation and the known increased risks for fetal pathology in euploid fetuses with increased NT measurements, serial ultrasounds were performed. At 30 weeks gestation prominent fetal bowel and possible mild ascites were noted on twin B. MCA-PSV on twin B was significantly elevated at 64 cm/s (1.60 MOM) corresponding to a hematocrit of 24% [4]. MCA-PSV for twin A was normal. Careful follow-up was elected, and fetal testing was reassuring. At 33 weeks 2 days gestation the finding of possible mild ascites had resolved but the MCA-PSV remained elevated at 73 cm/s (1.57 MOM) corresponding to a hematocrit of 24.9% [4]. Cordocentesis was elected to help with delivery planning and due to concern for potential fetal jeopardy due to unexplained anemia. Due to the late gestation and placental topology it was difficult to determine with certainty which placental cord insertion belonged to each twin, and so both were sampled. The findings are tabulated in Table 1. It was concluded that the relative microcytosis in twin B (93.3 fL, normal for 33 weeks = 113.6 fL) [5] most likely explained the falsely elevated MCA-PSV measurements, and was presumed secondary to *α*-thalassemia trait in that twin. The patient was managed expectantly, and spontaneously delivered uneventfully at 36 weeks. Hemoglobin electrophoresis for twin B done at 4 months of age was consistent with *α*-thalassemia trait (HbA 91.6%, HbA2 1.9%, HbF 6.5%). Both children were otherwise well at that time.

## 3. Comment

Alpha-thalassemia trait results from deletion of two of the four *α* globin genes, resulting in deficient *α* chain production and an associated microcytic anemia. In people of Asian ethnicity, the two missing *α* genes are typically arranged in cis (*α* *α*/− −), resulting in transmission of the trait to 50% of offspring.

In this case, serial ultrasounds led to a question of mild ascites and the finding of abnormal MCA Doppler velocimetry. In retrospect, it seems likely that the possible mild ascites was an artifact of heightened scrutiny related to the increased NT measurements. The abnormal MCA Doppler studies resulted in a concern for fetal anemia and possible fetal jeopardy and it was elected to obtain fetal hematocrits to guide further management. Cordocenteses wrer performed on both twins for technical reasons as discussed, providing a normal control for the abnormal results from twin B. The MCV on twin B was midway between the normal fetal MCV on twin A and the abnormally low MCV on the patient, and was in the normal range for adult hemoglobin.

This case provides a “natural experiment” confirming that MCA-PSV measurements may be raised by the presence of red blood cells with an adult MCV despite a normal hematocrit, as observed in fetuses that have been transfused with adult blood. In the case of elevated MCA-PSV resulted following fetal transfusion of adult RBC, the increased MCA-PSV measurements have been attributed to reduced posttransfusion viscosity due to both the smaller size of the transfused adult RBC and reduced cellular rigidity of adult RBC [6, 7]. Our results support the conclusion that the elevated MCA-PSV may be due to reduced MCV. Any role for altered cell membrane characteristics (due to intracellular accumulation of abnormal hemoglobin, e.g.,) is less clear.

Fetal *α*-thalassemia trait, and presumably other hemoglobinopathies resulting in relative fetal microcytosis, should be considered when unexpectedly elevated MCA-PSV results are obtained.

## Figures and Tables

**Table 1:** Maternal and fetal hematocrit and MCV values.

	Hematocrit (%)	MCV (fL)
Maternal	25.5	73.5
Twin A	40.1	109.8
Twin B	36.3	93.3
